# Autothermal Sorption-Enhanced Steam Reforming of Renewable Syngas: Composition-Dependent Hydrogen Yield and Energy Efficiency

**DOI:** 10.1021/acs.energyfuels.6c01902

**Published:** 2026-07-17

**Authors:** Alejandra Vega, Fernando Rubiera, Covadonga Pevida, María Victoria Gil

**Affiliations:** 16379 Institute of Carbon Science and Technology (INCAR), CSIC, Francisco Pintado Fe 26, Oviedo 33011, Spain

## Abstract

Producing renewable hydrogen from biomass is essential for the transition to low-carbon energy systems and sustainable fuel production. Biomass-derived syngas streams from biorefineries are a promising hydrogen source, but their compositional variability and high fractions of CO_2_ and H_2_ significantly affect hydrogen production performance. Sorption-enhanced steam reforming (SESR) combines steam reforming with in situ CO_2_ capture using CaO-based sorbents, enhancing hydrogen production through process intensification. This work investigates autothermal SESR enabled by CO_2_-rich feed compositions and heat integration to improve energy efficiency. An Aspen Plus equilibrium model was used to evaluate the effects of syngas composition and operating conditions (steam-to-carbon = 2.5–6.5, CaO/C = 1.5–2.5) on hydrogen production and energy performance at 600 °C and 10 bar. CO-rich feeds improve H_2_ purity, CH_4_ conversion, CO_2_ capture efficiency, and cold gas efficiency (CGE) by promoting the water–gas shift reaction and methane reforming through reduced CO_2_ partial pressure. In contrast, CH_4_- and H_2_-rich feeds decrease H_2_ purity and methane conversion due to lower effective steam-to-methane ratios and equilibrium limitations. However, these compositions improve overall energy efficiency by reducing the external fuel required for sorbent regeneration. Increasing steam availability enhances performance up to a threshold beyond which Ca­(OH)_2_ formation becomes significant, which strongly penalizes efficiency due to additional regeneration energy demand. Under optimal conditions, hydrogen purities up to 99.2 vol %, CO_2_ capture efficiency of 98.8%, and a CGE of 81% are achieved. These results help define operating windows for autothermal SESR and support the design of flexible and energy-efficient hydrogen production systems.

## Graphical abstract

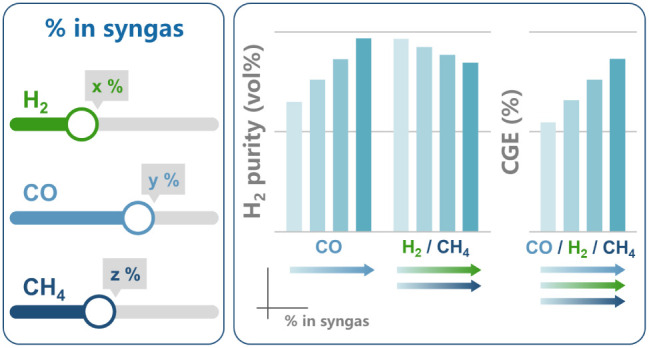

## 1 Introduction

In recent years, the urgent need to mitigate climate change, reduce greenhouse gas emissions, and ensure long-term energy security has intensified the global search for sustainable energy solutions.^1,2^ Among these, hydrogen has emerged as a clean, flexible, and high-energy-density fuel that is essential to the ongoing energy transition.^3^ Its high gravimetric energy content, zero emissions at the point of use, and applicability across multiple sectors, including transportation, power generation, chemical manufacturing, and metallurgy, highlight its strategic importance for achieving climate neutrality. Beyond serving as an energy carrier, hydrogen is also a key industrial feedstock for ammonia synthesis, methanol production, and hydrocracking processes.^4^ Furthermore, it is a critical enabler of CO_2_ valorization, supporting the synthesis of carbon-neutral fuels, such as e-fuels and advanced biofuels, which are indispensable for meeting global emission reduction targets. This dual functionality positions hydrogen as a crucial component of net-zero energy systems, enabling decarbonization while sustaining industrial productivity.

Currently, most hydrogen is produced from fossil sources, primarily through steam reforming (SR) of methane or natural gas, processes that are inherently associated with high CO_2_ emissions.^5,6^ Although water electrolysis offers a low-carbon alternative, its large-scale deployment remains constrained by the high electricity demand, limited availability of renewable power, and associated costs.^7,8^ As a result, renewable hydrogen production from biomass has attracted increasing attention, as it provides a carbon-neutral complement to electrolytic hydrogen while enabling the displacement of fossil-derived routes.^9,10^ Biomass includes a broad range of feedstocks, including agricultural residues, forestry waste, municipal solid waste, algae, and dedicated energy crops, which can be transformed into valuable energy carriers via biological or thermochemical processes such as fermentation, pyrolysis, and gasification.^11–14^ Among these, thermochemical conversion is interesting because it produces a versatile gaseous mixture, commonly known as syngas, composed mainly of H_2_, CO, CH_4_, and CO_2_. This syngas can serve as a renewable feedstock for reforming processes to produce high-purity hydrogen, thereby enabling the integration of hydrogen generation within biorefineries and supporting circular, carbon-neutral energy systems.

A defining characteristic of biomass as an energy resource is its inherent variability in composition, structure, and physicochemical properties. These variations arise from differences in feedstock origin, growth conditions, harvesting practices, and storage methods. Developing flexible conversion processes capable of accommodating this variability is, therefore, essential for achieving stable and efficient operation in biomass-based systems. Moreover, biomass availability is seasonal and geographically dispersed, highlighting the importance of feedstock flexibility for maintaining continuous operation in biorefineries. Furthermore, after thermochemical conversion, the resulting syngas streams remain compositionally dynamic, influenced by feedstock type,^15,16^ gasifying agent,^17–19^ and process conditions. For example, lignocellulosic biomass typically produces syngas richer in H_2_ and CO, whereas agricultural residues, which contain higher volatile fractions, tend to yield streams with greater CH_4_ fractions.^20–22^ These compositional variations are significant because they influence reaction equilibria, heat balances, and CO_2_ capture performance in downstream reforming processes, ultimately affecting hydrogen production and overall process efficiency.

Sorption-enhanced steam reforming (SESR) is a promising process intensification strategy to overcome the thermodynamic limitations of conventional steam reforming by incorporating in situ CO_2_ capture using solid sorbents such as CaO.^23,24^ SESR couples steam methane reforming (SMR) (eq 1), the water–gas shift (WGS) reaction (eq 2), and CaO carbonation (eq 3), thereby shifting the overall equilibrium toward enhanced hydrogen production according to Le Chatelier’s principle. The overall SESR reaction is shown in eq 4.

1 $${\mathrm{CH}}_{4}+H_{2}O⇄\mathrm{CO}+3H_{2},\Delta H_{r}^{{}^{\circ}}=+206\;\mathrm{kJ}\;{\mathrm{mol}}^{-1}$$

2 $$\mathrm{CO}+H_{2}O⇄{\mathrm{CO}}_{2}+H_{2},\Delta H_{r}^{{}^{\circ}}=-41\;\mathrm{kJ}{\;\mathrm{mol}}^{-1}$$

3 $${\mathrm{CaO}}_{\left(s\right)}+{\mathrm{CO}}_{2}⇄{\mathrm{CaCO}}_{3\left(s\right)},\Delta H_{r}^{{}^{\circ}}=-178\;\mathrm{kJ}\;\mathrm{mo}l^{-1}$$

4 $$CH_{4}+2H_{2}O+CaO_{\left(s\right)}⇄4H_{2}+CaCO_{3\left(s\right)},\Delta H_{r}^{{}^{\circ}}=-13\;kJ\;mol^{-1}$$

In situ CO_2_ removal enhances H_2_ yield and purity, reducing downstream separation requirements. Additionally, the exothermic carbonation reaction offsets the strong endothermic heat demand of steam reforming, thereby improving the process’s thermal efficiency compared with conventional SR.^25^ When processing CO_2_-containing feeds such as syngas, this benefit becomes even more pronounced, as the captured CO_2_ from the feed provides additional exothermic heat, further supporting the system’s energy balance. Therefore, detailed SESR simulations using syngas-like mixtures are essential to understand energy utilization under variable feed compositions and to quantify the additional exothermic contribution arising from CO_2_ in biomass-derived gases. Moreover, cyclic sorbent regeneration via CaCO_3_ calcination (the reverse of eq 3) is highly endothermic, posing significant challenges for efficient heat recovery and thermal integration in SESR systems. Efficient heat-exchanger network (HEN) design is therefore essential to recover exothermic heat released during carbonation and WGS reactions in the SESR reactor, reducing auxiliary energy demand and improving overall efficiency.^25,26^

Furthermore, operating SESR under elevated pressures offers several advantages for process intensification and system integration. Pressurization reduces reactor volume, decreases the need for downstream hydrogen compression, and facilitates direct coupling with purification technologies such as pressure swing adsorption (PSA).^27,28^ High-pressure hydrogen production is also compatible with numerous industrial applications, including Fischer–Tropsch synthesis,^29^ dimethyl ether production,^30^ hydrotreating,^31^ hydrogenation processes,^32^ and direct iron reduction in the steel sector.^33^ Beyond industrial use, pressurized hydrogen is essential for refueling stations for fuel cell vehicles,^34^ injection into natural gas pipelines,^35^ and large-scale storage and transport via tanks or underground caverns.^36^ However, operating SESR at elevated pressure also introduces important thermodynamic trade-offs. Increasing pressure shifts the steam reforming equilibrium (eq 1) toward the reactants, reducing hydrogen formation, while simultaneously enhancing CO_2_ capture through the carbonation reaction (eq 3).^37^ This opposing behavior complicates the identification of optimal operating windows. A further challenge in pressurized SESR is the formation of secondary solid phases, particularly Ca­(OH)_2_ via CaO hydration (eq 5),^38^ which consumes active sorbent and lowers the adequate CO_2_ capture capacity.

5 $${\mathrm{CaO}}_{\left(s\right)}+H_{2}O_{\left(g\right)}⇄\mathrm{Ca}{\left(\mathrm{OH}\right)}_{2\left(s\right)},\Delta H_{r}^{{}^{\circ}}=-109\;\mathrm{kJ}\;{\mathrm{mol}}^{-1}$$

Ca­(OH)_2_ formation is thermodynamically favored at intermediate temperatures (500–700 °C) and high steam partial pressures,^39,40^ conditions that depend strongly on both feed composition and operating pressure. However, the influence of syngas composition on SESR performance and energy efficiency under pressurized conditions remains insufficiently studied. Most previous investigations have relied on simplified or fixed feed compositions^41^ and were conducted at lower pressures.^26,41,42^ Consequently, a knowledge gap persists regarding SESR behavior with realistic biomass-derived syngas streams under pressurized conditions, particularly given the inherent variability in feed composition in biorefinery operations.

In this work, we develop a detailed Aspen Plus simulation framework to investigate the high-pressure SESR of biomass-derived gaseous streams. These feedstocks are rich in H_2_, CO, CH_4_, and CO_2_ and are increasingly available from emerging biorefineries and carbon-intensive industries implementing circular carbon strategies. The study aims to understand how variations in syngas composition influence SESR performance, including hydrogen production, CO_2_ capture, and overall energy efficiency. Particular attention is given to heat integration to recover the additional heat released by CO_2_ removal via the exothermic carbonation reaction, thereby minimizing external energy requirements. By quantifying key performance indicators, including hydrogen yield and purity, CH_4_ and CO conversion, cold gas efficiency (CGE), net efficiency (NE), and CO_2_ capture efficiency, this work establishes the thermodynamic and compositional limits that govern high-pressure SESR performance. The results aim to provide practical insights for designing energy-efficient SESR systems that can convert variable biomass-derived syngas into renewable hydrogen within carbon-neutral biorefineries.

## 2 Materials and Methods

### 2.1 Process Configuration and Model Development

An autothermal SESR process for hydrogen production from syngas, integrating steam reforming with in situ CO_2_ capture and sorbent regeneration, was developed in Aspen Plus V14 (AspenTech). An equilibrium-based thermodynamic model was used to determine the theoretical performance limits of the SESR process under different operating conditions and syngas compositions. The model assumes steady-state operation. Under optimal operating conditions, SESR systems have been shown to approach equilibrium due to the strong coupling between reforming, water–gas shift, and carbonation reactions, thus providing a meaningful upper bound for hydrogen production and CO_2_ capture.^23,24^

To maximize thermal efficiency, the model includes an integrated heat exchanger network (HEN) for optimized heat recovery. A simplified representation of the reference process configuration is shown in Figure 1, while the complete Aspen Plus flowsheet is presented in Figure 2. This configuration was used to evaluate the influence of syngas composition on SESR performance. The resulting framework enables the assessment of thermodynamic behavior, energy integration, and overall process efficiency under varying feed conditions.

**Figure 1:**
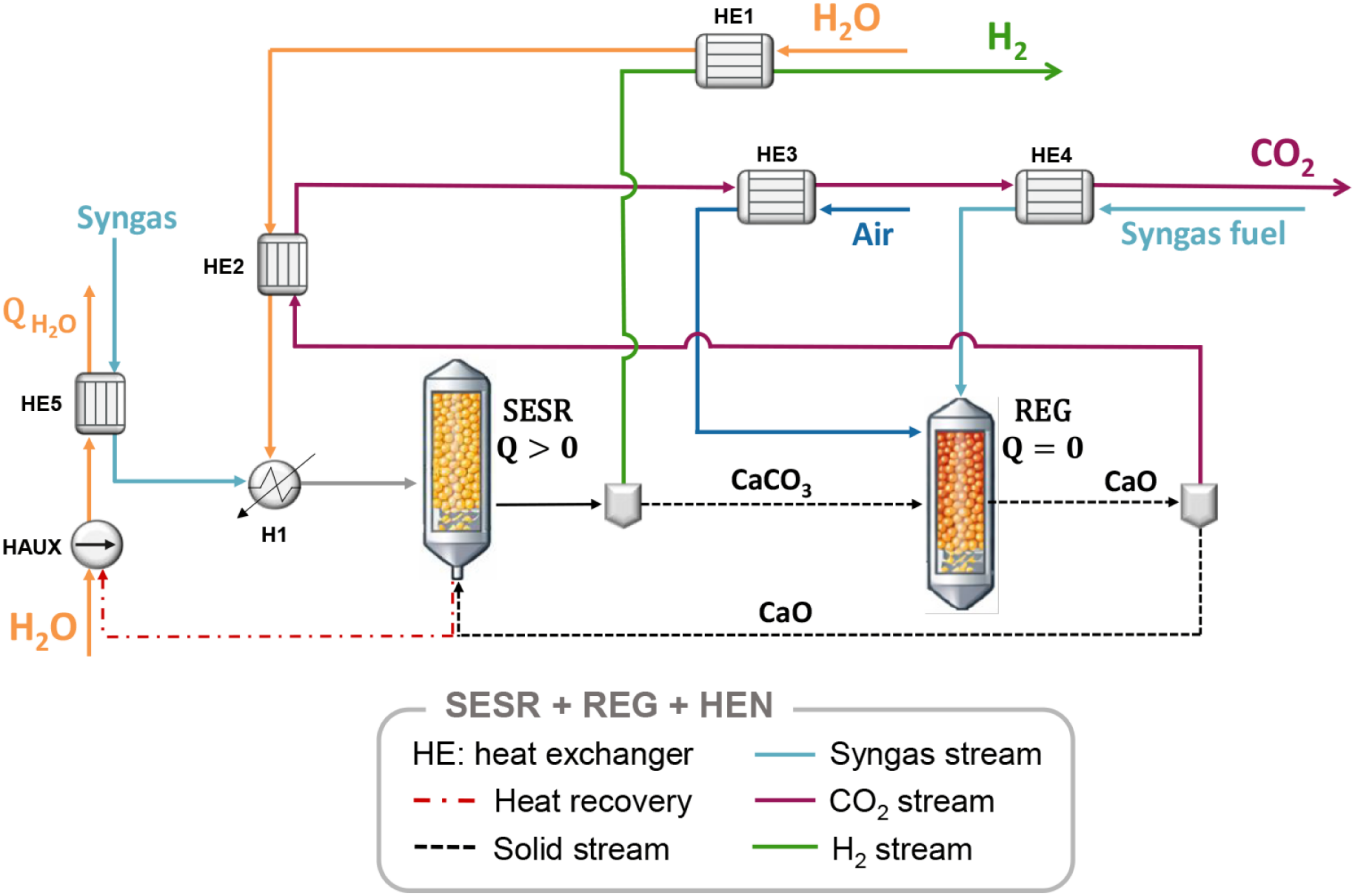
Conceptual diagram of the autothermal sorption-enhanced steam reforming (SESR) process for renewable H_2_ production from syngas, showing the main process units, material streams, and heat-integration strategy.

**Figure 2:**
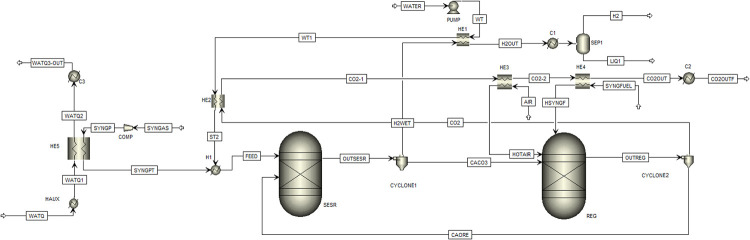
Aspen Plus simulation flowsheet of the autothermal syngas SESR process used in this study, including the reforming reactor, sorbent regeneration unit, and heat exchanger network (HEN).

The main thermodynamic and operational assumptions applied in the base model are summarized in Table 1. The solid sorbent was represented as pure CaO, and the feed gas was modeled as a mixture of H_2_, CO, CH_4_, and CO_2_. Feed composition was varied across a range representative of biomass-derived gaseous streams from gasification and pyrolysis processes. Steam-to-carbon (S/C) molar ratios of 2.5, 4.5, and 6.5, and CaO-to-carbon (CaO/C) molar ratios of 1.5, 2.0, and 2.5 were evaluated. Process simulations were conducted at 600 °C and 10 bar. This temperature was selected based on previous experimental work on SESR of biomass-derived syngas, which was identified as optimal for achieving high hydrogen yield while maintaining effective in situ CO_2_ capture.^23,24^ The complete set of operating variables used in the parametric study is shown in Table 2.

**Table 1:** Design Assumptions Used in the Base-Case SESR Simulation

Parameter	Value
Water feed inlet temperature (°C)	25
Water feed inlet pressure (bar)	1
Reformer pressure (bar)	10
Reformer temperature (°C)	600
Reformer heat loss (%)	10
Calciner temperature (°C)	800
Calciner pressure (bar)	1
Excess oxygen in calciner (%)	5
Air inlet temperature (°C)	25
Air pressure (bar)	1
Fuel feed inlet temperature (°C)	25
Fuel feed pressure (bar)	1
Calcination conversion (%)	100
Heat exchanger pinch (°C)	20
Isentropic efficiency of compressors and water pump efficiency (%)	83
Mechanical efficiency of compressors and water pump (%)	98

**Table 2:** Operating Variables Considered in the Parametric Simulation Study

Parameter	Value
H_2_ in feed gas (vol %)	0–50
CH_4_ in feed gas (vol %)	10–30
CO in feed gas (vol %)	10–40
CO_2_ in feed gas (vol %)	0–50
Steam/C molar ratio (S/C)	2.5–6.5
CaO/C molar ratio (CaO/C)	1.5–2.5

The process configuration consists of two main reactors. In the SESR reactor, hydrogen is generated through simultaneous steam reforming (eq 1), the WGS reaction (eq 2), and in situ CO_2_ capture via CaO carbonation (eq 3). Continuous CO_2_ removal shifts the reaction equilibria toward increased hydrogen production, according to Le Châtelier’s principle. To approach autothermal behavior, the model incorporates heat recovery from the exothermic heat released during SESR. An overall heat-transfer loss of 10% was assumed, consistent with reported efficiencies for reverse-flow reactors used in exothermic systems.^25,43^ Feasible heat-integration strategies include fluidized-bed heat exchangers with immersed tubes^44,45^ and heat-pipe-based systems, which have been proposed for indirect heat transfer in chemical looping calciners^46–48^ and sorption-enhanced steam methane reforming (SESMR) units.^25,26^ This configuration provides a realistic thermodynamic basis for evaluating energy recovery in SESR under the operating conditions studied. In the Aspen Plus model, heat recovery is explicitly represented through an auxiliary water stream (WATQ) that circulates through heat exchanger HE5, which absorbs the heat released in the SESR reactor, and reintroduces it into the process (Figure 2). A design specification adjusts the WATQ flow rate according to the excess heat produced by the SESR reactor and its temperature. An auxiliary heater (HAUX) is included to simulate the reuse of this internally recovered heat.

The regeneration reactor (REG) receives carbonated sorbent (CaCO_3_) from the SESR stage and regenerates it via endothermic calcination (reverse carbonation, eq 3) at 800 °C and 1 bar,^49^ ensuring complete sorbent regeneration. Heat is supplied by direct combustion of syngas with the same composition as the SESR feed. Compared with indirect heating methods, direct combustion offers a more straightforward and efficient approach to meeting the substantial thermal demand of calcination.^50,51^ Combustion is carried out with air as the oxidant. A 5% excess of oxygen is used to ensure complete oxidation and prevent the formation of incomplete oxidation products (CO, H_2_, or elemental carbon) in the regenerator outlet.^25,26,52^ Design specifications automatically adjust the syngas flow rate to the REG reactor to the minimum needed to satisfy the calcination duty.

CaO-based sorbents progressively lose activity over repeated carbonation–calcination cycles due to sintering and other structural degradation mechanisms.^53^ To represent this reduced reactivity, consistent with values reported in cyclic SESR experiments,^54,55^ an excess sorbent inventory was employed in the simulations. This approach reflects industrial practice, where sorbent deactivation is compensated through sorbent recirculation and the addition of fresh makeup sorbent to maintain the required CO_2_ capture capacity. In practical operation, the circulation rates of fresh and regenerated sorbent are adjusted to maintain a stable balance between capture and regeneration. The CaO/C molar ratio was defined as the ratio of circulating CaO (exchanged between the SESR and REG reactors) to the total carbon content in the syngas feed (including CO, CH_4_, and CO_2_). The selected CaO/C range (1.5–2.5) allowed evaluation of the impact of sorbent inventory on the formation of secondary solid phases, particularly Ca­(OH)_2_. Under elevated pressures and intermediate temperatures, excess CaO combined with high steam availability can promote CaO hydration, reducing the effective CO_2_ capture and negatively affecting overall reforming performance. In addition, the CaO/C ratio also influences the thermal behavior of the system through the total sorbent inventory, which determines the sensible heat required for heating and circulating the solid phase within the process.

Both the SESR and REG reactors were modeled using RGibbs equilibrium blocks, which determine outlet compositions by minimizing the total Gibbs free energy—a widely used approach for SESR simulations.^25,26,41,52^ The species considered in the equilibrium calculations included H_2_, CH_4_, CO, CO_2_, H_2_O, O_2_, N_2_, CaO, Ca­(OH)_2_, CaCO_3_, C_2_H_4_, C_2_H_6_, and solid carbon (graphite, included to account for potential coke formation). The latter three species showed negligible equilibrium concentrations under all of the operating conditions evaluated. Thermodynamic properties were calculated using the Peng–Robinson equation of state, which is appropriate for high-temperature, multicomponent gas–solid systems.

The heat exchanger network (HEN) was designed to maximize internal heat recovery while minimizing the number of units. It preheats the reactants and generates the steam required for reforming, thereby reducing the need for external energy input. Water for steam production is first preheated in HE1 using the hydrogen-rich stream exiting the SESR reactor (a 5 °C superheat margin is maintained at the hot-stream outlet to avoid condensation). The water is then further heated in HE2 by recovering heat from the CO_2_-rich stream leaving the REG reactor. The resulting steam is mixed with the syngas feed in mixer H1 to reach the target SESR inlet temperature. A design specification initially estimates the heat duty required in H1 to achieve the desired reforming temperature. In contrast, a second specification drives the final duty of H1 to zero by adjusting the hot-side temperature of HE2. This approach ensures that H1 operates strictly as a thermal mixer without external heating.

Residual heat in the CO_2_-rich stream is further recovered to preheat the REG reactor feed (i.e., the air and syngas used as fuel) using a design-defined temperature approach that maximizes heat recovery. The hydrogen-rich product stream is then cooled to 25 °C in cooler C1, enabling water condensation and subsequent removal in separator SEP1 (Figure 2). The CO_2_-rich product stream is also cooled to 25 °C in cooler C2 (Figure 2). Both resulting streams are suitable for downstream purification, compression, or storage. Auxiliary equipment, including compressors and pumps, is included to control flow rates and pressures of the water and air streams. Isentropic and mechanical efficiencies of 83% and 98%, respectively, are assumed, in accordance with literature values.^25,26^

### 2.2 Data Evaluation

The thermodynamic performance of the SESR process was assessed using the H_2_ yield, H_2_ purity, CO and CH_4_ conversions, and CH_4_ concentration in the outlet gas. These metrics were calculated using eqs 6–9.

6 $$H_{2}\;\mathrm{yield}\;(\%)=100\times \left[\frac{F_{H_{2},\mathrm{out}}}{F_{H_{2},\mathrm{in}}+F_{\mathrm{CO},\mathrm{in}}+4F_{{\mathrm{CH}}_{4},\mathrm{in}}}\right]$$

7 $$H_{2}\;\mathrm{purity}\;(\mathrm{vol}\;\%)=100\times \left[\frac{F_{H_{2},\mathrm{out}}}{\sum_{i}F_{i,\mathrm{out}}}\right]$$

8 $$CO/CH_{4}\;conversion\;(\%)=100\times [\frac{F_{CO/{\mathrm{CH}}_{4},in}-F_{CO/{\mathrm{CH}}_{4},out}}{F_{CO/{\mathrm{CH}}_{4},in}}]$$

9 $${\mathrm{CH}}_{4}\;\left(\mathrm{vol}\;\%\right)=100\times \left[\frac{F_{{\mathrm{CH}}_{4},\mathrm{out}}}{\sum_{i}F_{i,\mathrm{out}}}\right]$$

where *F*_*i*,in_ and *F*_*i*_,_out_ represent the inlet and outlet molar flow rates of species *i*, respectively, in the SESR reactor. The H_2_ yield quantifies the fraction of hydrogen obtained relative to the theoretical maximum based on SESR stoichiometry.

The system boundary includes the SESR reactor, regeneration reactor, heat-integration network, and auxiliary equipment required for operation, while excluding upstream feedstock production and downstream CO_2_ handling. To assess the system’s energy performance, the cold gas efficiency (CGE), net efficiency (NE), and fuel demand in the regeneration reactor were quantified using eqs 10–12. CGE represents the ratio of the chemical energy of the produced hydrogen and the recoverable process heat to the total thermal energy supplied to the process, including fuel consumed in both the SESR and REG reactors. NE further incorporates the electricity requirements of auxiliary equipment and accounts for thermal-to-electric conversion efficiency.

10 $$\mathrm{CGE}\;\left(\%\right)=100\times \left[F_{H_{2},\mathrm{out}}\cdot {\mathrm{LHV}}_{H_{2}}+Q_{\mathrm{recov}}]/[\left(F_{H_{2},\mathrm{in}}+F_{H_{2},\mathrm{add}}\right)\cdot {\mathrm{LHV}}_{H_{2}}+\left(F_{{\mathrm{CH}}_{4},\mathrm{in}}+F_{{\mathrm{CH}}_{4},\mathrm{add}}\right)\cdot {\mathrm{LHV}}_{{\mathrm{CH}}_{4}}+\left(F_{\mathrm{CO},\mathrm{in}}+F_{\mathrm{CO},\mathrm{add}}\right)\cdot {\mathrm{LHV}}_{\mathrm{CO}}\right]$$

11 $$\mathrm{NE}\;\left(\%\right)=100\times \left[F_{H_{2},\mathrm{out}}\cdot {\mathrm{LHV}}_{H_{2}}+Q_{\mathrm{recov}}]/[\left(F_{H_{2},\mathrm{in}}+F_{H_{2},\mathrm{add}}\right)\cdot {\mathrm{LHV}}_{H_{2}}+\left(F_{{\mathrm{CH}}_{4},\mathrm{in}}+F_{{\mathrm{CH}}_{4},\mathrm{add}}\right)\cdot {\mathrm{LHV}}_{{\mathrm{CH}}_{4}}+\left(F_{\mathrm{CO},\mathrm{in}}+F_{\mathrm{CO},\mathrm{add}}\right)\cdot {\mathrm{LHV}}_{\mathrm{CO}}+\frac{P_{e}}{\eta_{\mathrm{elect}}}\right]$$

12 $$\mathrm{Fraction}\;\mathrm{of}\;\mathrm{fuel}\;\mathrm{in}\;\mathrm{REG}\;\left(\%\right)=100\times \left(\frac{F_{\mathrm{syngas fed to REG}}}{F_{\mathrm{syngas fed to SESR}}+F_{\mathrm{syngas fed to REG}}}\right)$$

where LHV_*i*_ is the lower heating value of species *i* (242, 283, and 800 MJ kmol^–1^ for H_2_, CO, and CH_4_, respectively), *F*_*i*_,_add_ represents the additional molar flow rate of species *i* supplied to the REG reactor, *P*_e_ represents the auxiliary electricity consumption, and η_elect_ is the thermal-to-electric conversion efficiency, assumed to be 50%. The term *Q*_recov_ refers to the recoverable process heat, which is included in the efficiency metrics because it contributes to the system’s usable energy output. After full heat integration, any excess thermal energy remaining in the auxiliary water stream is stored as high-temperature steam. Since steam is an efficient thermal carrier, this energy can be reused for process heating or other high-temperature applications, or reutilized within the plant as process steam, thereby reducing the need for additional steam generation. To reflect this in the process evaluation, the remaining thermal energy in the auxiliary water stream is quantified and incorporated as recoverable process heat in the efficiency calculations.

Finally, the CO_2_ capture efficiency was defined as the ratio of CO_2_ captured to the total carbon entering the system (including CO, CO_2_, and CH_4_ in the feed and the additional fuel). It was calculated using eq 13, where F_CO2,REG_ is the outlet molar flow rate of CO_2_ from the REG reactor.

13 $${\mathrm{CO}}_{2}\;\mathrm{capture}\;\mathrm{efficiency}\;\left(\%\right)=100\times \left(\frac{F_{{\mathrm{CO}}_{2,\mathrm{REG}}}}{F_{{\mathrm{CO}}_{2,\mathrm{in}}}+F_{{\mathrm{CO}}_{2,\mathrm{add}}}+F_{{\mathrm{CH}}_{4,\mathrm{in}}}+F_{{\mathrm{CH}}_{4,\mathrm{add}}}+F_{{\mathrm{CO}}_{\mathrm{in}}}+F_{{\mathrm{CO}}_{\mathrm{add}}}}\right)$$

## 3 Results and Discussion

### 3.1 Effect of the CaO/C Molar Ratio on Ca­(OH)_2_ Formation in High-Pressure SESR

The formation of secondary solid phases, particularly Ca­(OH)_2_, can be a critical factor influencing both H_2_ production and CO_2_ capture efficiency when CaO is used as a sorbent in SESR.^38^ The CaO/C ratio is therefore a key operating parameter, especially under high-pressure conditions where competing reactions become strongly sensitive to gas composition. Ca­(OH)_2_ forms via the hydration reaction (eq 5), which can compete with carbonation (eq 3) for the available CaO. Before assessing the influence of syngas composition on SESR performance, the effect of the CaO/C ratio on Ca­(OH)_2_ formation was evaluated using syngas-like mixtures. Figure 3 shows the equilibrium fractions of Ca­(OH)_2_, CaO, and CaCO_3_ for SESR operated at 600 °C and 10 bar with S/C = 4.5, using feeds containing 30 vol % H_2_, 10 vol % CH_4_, CO varying from 10–40 vol %, and CO_2_ as the balance gas.

**Figure 3:**
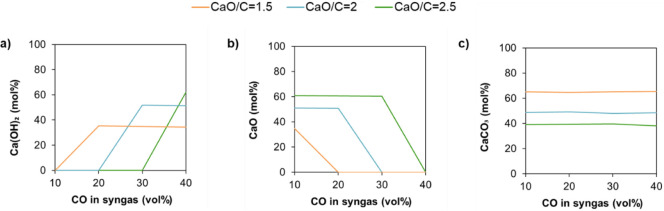
Effect of the CaO-to-carbon ratio (CaO/C) on the equilibrium distribution of solid sorbent phases during SESR: (a) Ca­(OH)_2_, (b) CaO, and (c) CaCO_3_. Three CaO/C ratios (1.5, 2.0, 2.5) were evaluated. Simulations were carried out at 600 °C, 10 bar, and S/C = 4.5 for syngas feeds containing 30 vol % H_2_, 10 vol % CH_4_, 10–40 vol % CO, and CO_2_ as the balance.

Figure 3a shows distinct patterns of Ca­(OH)_2_ formation as a function of the CaO/C ratio. At low CO contents, no Ca­(OH)_2_ is observed, but hydration progressively appears as CO increases. At a fixed CaO/C ratio (and therefore fixed H_2_O/CaO, since S/C is constant), the main variable is the CO_2_ partial pressure. As CO increases, the CO_2_ content in the feed decreases (since CO_2_ is the balancing gas), reducing the CO_2_/CaO ratio and weakening the driving force for carbonation. As carbonation becomes less favored, more free CaO remains available to react with steam, initiating the formation of Ca­(OH)_2_.

As the CaO/C ratio increases, the onset of Ca­(OH)_2_ formation shifts toward higher CO fractions in the feed (i.e., lower CO_2_ levels). At a fixed S/C, increasing CaO/C raises the amount of CaO relative to steam, effectively decreasing the H_2_O/CaO ratio. When the steam per mole of CaO becomes limited, CaO hydration becomes less favorable, and carbonation remains the dominant solid-phase reaction. Under these conditions, Ca­(OH)_2_ can form only when the CO_2_ levels have dropped sufficiently for a substantial fraction of CaO to remain uncarbonated. As a result, Ca­(OH)_2_ formation is displaced toward syngas compositions containing higher CO concentrations.

Therefore, Ca­(OH)_2_ formation requires two conditions to occur simultaneously: a sufficiently low CO_2_ partial pressure (to prevent carbonation from consuming CaO) and sufficient steam per mole of CaO (to drive hydration). At low CaO/C ratios, the higher H_2_O/CaO ratio enables hydration even at moderate CO_2_ levels. In contrast, at higher CaO/C, the lower H_2_O/CaO ratio delays hydration until CO_2_ is very low. For example, at 30 vol % CO, Ca­(OH)_2_ forms at CaO/C = 1.5–2.0 but not at CaO/C = 2.5, when the larger CaO inventory reduces the steam availability per CaO, allowing carbonation to dominate over a wider range of feed compositions. Overall, high H_2_O/CaO ratios promote hydration, particularly when CO_2_ partial pressure is low and sufficient uncarbonated CaO remains, whereas high CO_2_/CaO ratios favor carbonation and suppress hydration under steam-limited conditions.

Figure 3b shows that whenever Ca­(OH)_2_ forms, free CaO disappears from the solid phase. Consistently, Figure 3c indicates that under these conditions, the remaining CaO is almost entirely converted to CaCO_3_ via carbonation. When hydration occurs, CaCO_3_ levels decrease slightly, confirming that Ca­(OH)_2_ formation reduces the amount of CaO available for CO_2_ capture. This behavior illustrates the competition between carbonation and hydration: carbonation dominates at higher CO_2_ partial pressures and limited steam, whereas hydration becomes significant at high H_2_O/CaO ratios, reducing the effective CO_2_ capture under steam-rich conditions.

For the subsequent analysis of the syngas composition effect, a CaO/C ratio of 2.5 was selected, as it minimizes Ca­(OH)_2_ formation under the studied conditions, providing greater flexibility to study different operating conditions. Using CaO in excess of the stoichiometric requirement is common practice in systems employing Ca-based sorbents, since it compensates for incomplete carbonation, kinetic limitations, and gradual sorbent deactivation over multiple cycles.^26^

### 3.2 Effect of Syngas Composition on High-Pressure SESR Performance and Energy Efficiency

#### 3.2.1 Effect of CO Content in Syngas

The effect of CO concentration in the syngas feed was assessed between 10–40 vol % (with 30 vol % H_2_, 10 vol % CH_4_, and CO_2_ as the balance), at 600 °C, 10 bar, and CaO/C = 2.5, for S/C ratios of 2.5, 4.5, and 6.5. Figure 4 summarizes the influence of CO content on SESR performance and energy efficiency. As shown in Figure 4a, at fixed S/C, Ca­(OH)_2_ formation is favored as CO content increases. A higher CO level decreases CO_2_ in the feed, lowering the CO_2_/CaO ratio and reducing the driving force for carbonation. Consequently, more unreacted CaO remains available for hydration. This trend is consistent with experimental studies showing that Ca­(OH)_2_ formation can occur in CaO-based sorbents exposed to humid gas streams during carbonation–calcination cycling, where steam availability plays a critical role in determining conversion and hydrogen production.^40,56^

**Figure 4:**
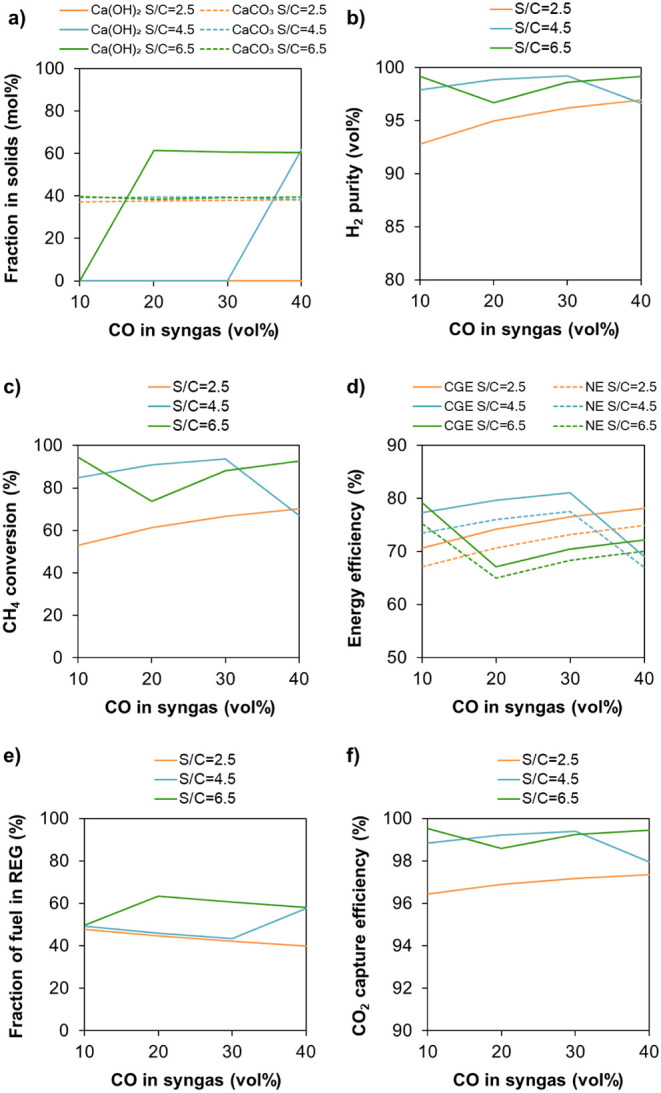
Effect of CO content in the syngas on SESR performance: (a) solid-phase distribution (Ca­(OH)_2_ and CaCO_3_), (b) H_2_ purity, (c) CH_4_ conversion, (d) cold gas efficiency (CGE) and net efficiency (NE), (e) regeneration fuel demand, and (f) CO_2_ capture efficiency. Three steam-to-carbon ratios (S/C = 2.5, 4.5, 6.5) are compared. Conditions: 600 °C, 10 bar, CaO/C = 2.5, syngas = 30 vol % H_2_, 10 vol % CH_4_, 10–40 vol % CO, and CO_2_ as the balance.

Higher S/C ratios promote hydration by increasing steam availability. At low CO (10 vol %), no hydration occurs at any S/C because carbonation dominates (Figure 4a). At intermediate CO (20–30 vol %), with lower CO_2_ levels, hydration appears only at S/C = 6.5, where the steam partial pressure is highest. At 40 vol % CO, the further reduction in CO_2_ partial pressure allows hydration to occur already at S/C = 4.5. Consequently, Figure 4a shows that no Ca­(OH)_2_ formation occurs at S/C = 2.5 for any CO level, while hydration occurs at 40 vol % CO for S/C = 4.5 and at ≥20 vol % for S/C = 6.5. Thus, increasing S/C shifts the onset of CaO hydration to lower CO contents, which is consistent with thermochemical trends reported for SESR of oxygenated hydrocarbons, where high steam partial pressures promoted hydration while hindering carbonation.^57^

When Ca­(OH)_2_ does not form (all CO levels at S/C = 2.5 and ≤30 vol % CO at S/C = 4.5), SESR performance improves with increasing CO. Figure 3b–c shows that both H_2_ purity and CH_4_ conversion increase because higher CO enhances the WGS reaction, while lower CO_2_ in the feed increases the thermodynamic driving force for reforming. Once hydration initiates (40 vol % CO at S/C = 4.5 and ≥20 vol % CO at S/C = 6.5), both H_2_ purity and CH_4_ conversion decline sharply, as does H_2_ yield (Figure S1a). In contrast, CO conversion remains nearly constant (∼99.8–99.9%, Figure S1b), indicating that CO conversion is not rate-limiting under the studied conditions. These results show that CaO hydration primarily decreases methane reforming by reducing the available steam, as evidenced by the strong increase in the outlet CH_4_ concentration (Figure S1c), while the WGS reaction continues to operate near equilibrium even during hydration.

The energy-related indicators, i.e., cold gas efficiency (CGE) and net efficiency (NE), follow the same overall behavior as H_2_ purity and CH_4_ conversion (Figure 4d), decreasing sharply when Ca­(OH)_2_ forms. NE is consistently lower than CGE because it accounts for auxiliary electricity demand. In the absence of hydration (e.g., 10 vol % CO), both efficiencies increase with S/C, with a great improvement from S/C = 2.5 to 4.5 and only marginal gains from S/C = 4.5 to 6.5. However, at 40 vol % CO for S/C = 4.5 and ≥20 vol % CO for S/C = 6.5, Ca­(OH)_2_ formation (Figure 4a) causes a pronounced drop in efficiency, leading to values even lower than those at S/C = 2.5. These results highlight the detrimental effect of Ca­(OH)_2_ on overall energy performance, consistent with CaO-based sorption-enhanced reforming studies of biogas, where hydration under steam-rich conditions was also reported to reduce H_2_ yield and thermal efficiency.^26^ Nevertheless, although S/C = 2.5 gives the highest energy efficiencies (Figure 4d), it also results in comparatively low H_2_ purity (∼96 vol %) (Figure 4b).

Figure 3d further shows that at S/C = 2.5, where hydration is absent, increasing CO content improves both CGE and NE. This arises because higher CO reduces the need for external fuel in the regeneration step, as a larger fraction of the feed’s chemical energy becomes available to drive sorbent regeneration. At higher S/C, regeneration fuel demand remains close to that at S/C = 2.5 (Figure 4e), as long as no Ca­(OH)_2_ forms (e.g., 10 vol % CO at S/C = 6.5 or 10–30 vol % CO at S/C = 4.5, Figure 4a). Once hydration occurs, however, fuel consumption rises sharply because regeneration must supply the heat for both CaCO_3_ calcination and Ca­(OH)_2_ decomposition. This additional decomposition requirement substantially increases the overall regeneration energy demand and directly contributes to the reduction in CGE and NE.

CO_2_ capture efficiency (Figure 4f) increases with CO content in the absence of Ca­(OH)_2_ (e.g., S/C = 2.5), as enhanced WGS activity and higher CH_4_ conversion produce more CO_2_ available for capture. When hydration occurs, the effective CaO inventory decreases while CH_4_ conversion drops, reducing both the amount of CO_2_ generated and the sorbent’s uptake. Capture efficiency consequently declines, following the same trend observed for CH_4_ conversion. Consistently, the outlet CH_4_ concentration shows an inverse relationship with CO_2_ capture efficiency (Figure S1c).

#### 3.2.2 Effect of CH_4_ Content in Syngas

The influence of CH_4_ concentration in the syngas feed was evaluated between 10 and 30 vol % (with 30 vol % H_2_, 30 vol % CO, and CO_2_ as balance) at 600 °C, 10 bar, and CaO/C = 2.5, for S/C ratios of 2.5, 4.5, and 6.5. Figure 5 summarizes the effect of CH_4_ variation on the main SESR performance indicators. As previously observed for feeds containing 30 vol % CO and 10 vol % CH_4_, Ca­(OH)_2_ forms only at S/C = 6.5 due to the high steam availability (Figure 4a). This behavior is confirmed in Figure 5a for higher CH_4_ contents, where no Ca­(OH)_2_ forms at S/C = 2.5 or 4.5, even though the associated decrease in CO_2_ would thermodynamically favor hydration. This is explained by the fact that higher CH_4_ concentrations intensify steam methane reforming, which consumes steam and reduces the amount available for CaO hydration.

**Figure 5:**
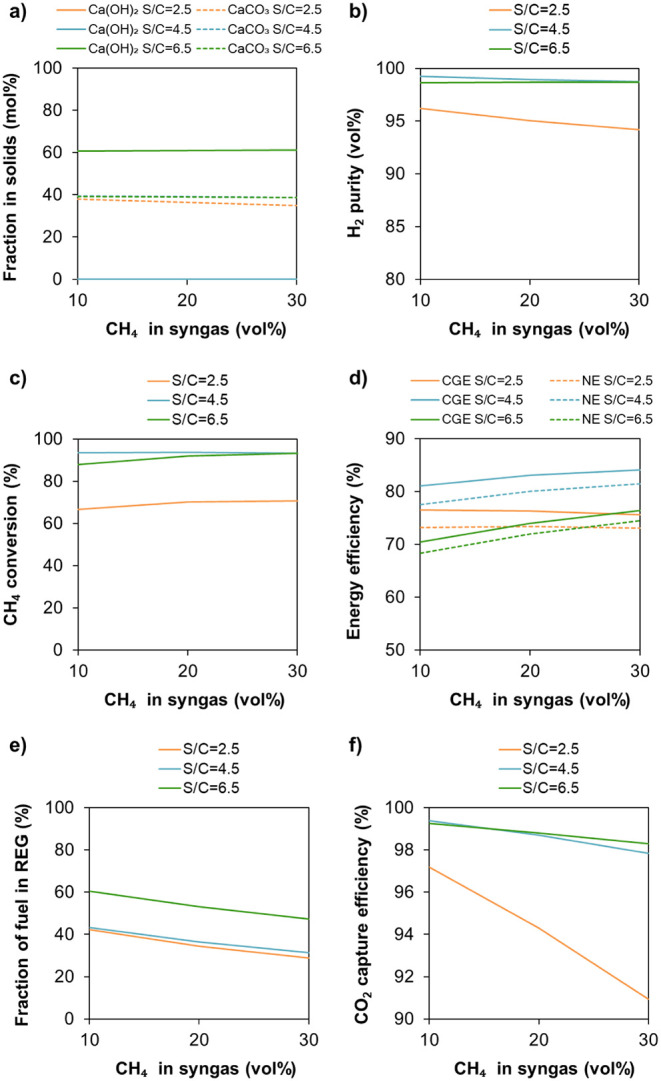
Effect of CH_4_ content in the syngas on SESR performance: (a) solid-phase distribution (Ca­(OH)_2_ and CaCO_3_), (b) H_2_ purity, (c) CH_4_ conversion, (d) cold gas efficiency (CGE) and net efficiency (NE), (e) regeneration fuel demand, and (f) CO_2_ capture efficiency. Three steam-to-carbon ratios (S/C = 2.5, 4.5, 6.5) are compared. Conditions: 600 °C, 10 bar, CaO/C = 2.5, syngas = 30 vol % H_2_, 30 vol % CO, 10–30 vol % CH_4_, and CO_2_ as the balance.

At S/C = 2.5 and 4.5, when Ca­(OH)_2_ formation is absent, increasing CH_4_ content leads to a slight decrease in H_2_ purity (Figure 5b) and H_2_ yield (Figure S2a). CH_4_ conversion remains nearly constant or decreases slightly (Figure 5c). Although more CH_4_ promotes the reforming reaction, the larger CH_4_ fraction reduces its conversion because, at fixed S/C, the amount of steam available per mole of CH_4_ decreases, limiting the reforming extent. Additionally, even when conversion remains similar, higher inlet CH_4_ results in higher outlet CH_4_ (Figure S2c), thereby lowering H_2_ purity. H_2_ purity is lowest at S/C = 2.5 (94.2–96.2 vol %) due to limited steam availability, while higher values (98.7–99.2 vol %) are attained at S/C = 4.5. At S/C = 6.5, H_2_ purity remains around ∼98.7 vol % (Figure 5a) because Ca­(OH)_2_ formation decreases CH_4_ conversion (Figure 5c). As in the CO variation study, CO conversion remains essentially constant (∼99.9%) across all CH_4_ levels (Figure S2b), confirming that the WGS reaction is not rate-limiting under the investigated conditions.

Energy efficiency trends (Figure 5d) show that the highest CGE and NE values are obtained at S/C = 4.5. Both metrics decrease noticeably at an S/C of 6.5 due to the formation of Ca­(OH)_2_. As the CH_4_ content increases, CGE and NE rise because the feed supplies more chemical energy, thereby reducing the external fuel required for sorbent regeneration (Figure 5e). Similar efficiency gains with higher CH_4_ contents have been reported in SESR simulations of biogas.^26^ However, once Ca­(OH)_2_ forms (at S/C = 6.5), the regeneration fuel demand increases sharply because additional heat is needed to decompose both CaCO_3_ and Ca­(OH)_2_, significantly raising the overall energy requirement.

Finally, the CO_2_ capture efficiency (Figure 5f) decreases as the CH_4_ content increases, following the same trend observed for H_2_ purity. Higher CH_4_ in the feed reduces the effective steam-to-CH_4_ ratio and limits reforming, leading to lower H_2_ production and, consequently, lower CO_2_ generation. This decrease in the level of CO_2_ formation directly reduces the capture efficiency. Consistently, CO_2_ capture efficiency shows an inverse trend to the outlet CH_4_ concentration (Figure S2c).

#### 3.2.3 Effect of H_2_ Content in Syngas

The influence of H_2_ concentration in the syngas feed was evaluated between 0 and 50 vol % (30 vol % CO, 10 vol % CH_4_, and CO_2_ as the balance) at 600 °C, 10 bar, and CaO/C = 2.5, for S/C ratios of 2.5, 4.5, and 6.5. The results are shown in Figure 6. Figure 6a shows that no Ca­(OH)_2_ forms at S/C = 2.5 across the entire H_2_ range. At S/C = 4.5, hydration begins once the feed contains ≥40 vol % H_2_, while at S/C = 6.5, Ca­(OH)_2_ forms for all H_2_ levels. This behavior arises because increasing H_2_ reduces CO_2_ (the balance gas), lowering the CO_2_/CaO ratio and favoring hydration over carbonation. Likewise, higher S/C further promotes hydration by increasing the H_2_O/CaO ratio, shifting its onset to lower H_2_ contents.

**Figure 6:**
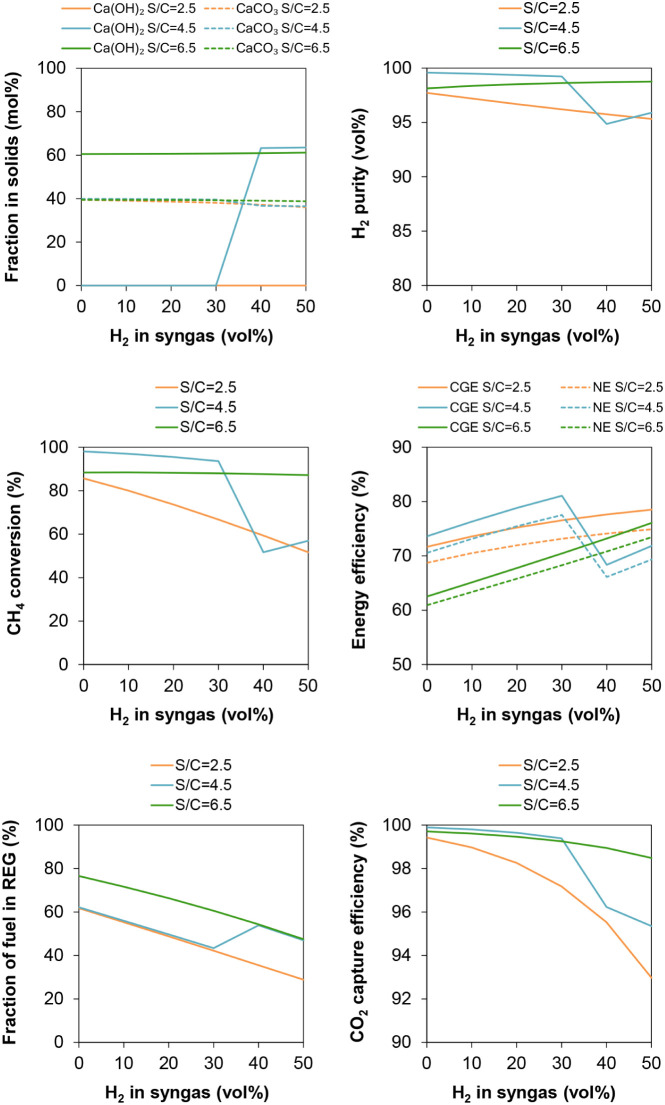
Effect of H_2_ content in the syngas on SESR performance: (a) solid-phase distribution (Ca­(OH)_2_ and CaCO_3_), (b) H_2_ purity, (c) CH_4_ conversion, (d) cold gas efficiency (CGE) and net efficiency (NE), (e) regeneration fuel demand, and (f) CO_2_ capture efficiency. Three steam-to-carbon ratios (S/C = 2.5, 4.5, 6.5) are compared. Conditions: 600 °C, 10 bar, CaO/C = 2.5, syngas = 30 vol % CO, 10 vol % CH_4_, 0–50 vol % H_2_, and CO_2_ as the balance.

When Ca­(OH)_2_ does not form (i.e., at S/C = 2.5 and at S/C = 4.5 for H_2_ ≤ 40 vol %; Figure 6a), increasing the H_2_ content progressively deteriorates SESR performance, as evidenced by the decrease in H_2_ purity (Figure 6b) and CH_4_ conversion (Figure 6c), due to the inhibitory effect of H_2_ on steam methane reforming and the WGS reaction. This effect is more pronounced at S/C = 2.5, indicating that additional steam at S/C = 4.5 partially compensates for the negative impact of H_2_. Similar reductions in CH_4_ conversion at elevated feed H_2_ levels have been reported in previous SESR studies.^58^

Once Ca­(OH)_2_ forms, the decline in performance becomes more pronounced. At S/C = 4.5, both H_2_ purity (Figure 6b) and CH_4_ conversion (Figure 6c) drop sharply between 30–40 vol % H_2_. At S/C = 6.5, where hydration occurs across the entire H_2_ range, both parameters remain below those at S/C = 4.5 up to 30 vol % H_2_. Above that point, H_2_ purity increases slightly due to the direct addition of H_2_ to the feed. A higher H_2_ content also reduces the fraction of carbon-containing species, lowering the CaO flow required to maintain CaO/C = 2.5. The smaller circulating sorbent mass flow reduces the steam consumed in Ca­(OH)_2_ formation. As a result, steam availability increases with higher feed H_2_, which can help to maintain or slightly increase CH_4_ conversion, as observed at 50 vol % H_2_ for S/C = 4.5 (Figure 6c). Similar behavior is observed for H_2_ yield (Figure S3a), while CH_4_ concentration (Figure S3c) follows an inverse trend. CO conversion remains nearly constant (∼99.9%, Figure S3b), confirming that the WGS reaction is not rate-limiting during hydration.

In the absence of Ca­(OH)_2_ formation (i.e., at S/C = 2.5 and at S/C = 4.5 for ≤30 vol % H_2_ (Figure 5b)), both CGE and NE increase with rising H_2_ content (Figure 6d). This improvement results from the lower fuel demand during sorbent regeneration (Figure 6e), since a larger fraction of the feed is directly combustible H_2_. In addition, because higher H_2_ in the feed reduces the carbon entering the system and thus the required CaO flow rate, the resulting decrease in circulating sorbent mass further lowers regeneration energy consumption, improving overall efficiency. Under these conditions, increasing S/C from 2.5 to 4.5 also improves efficiency because the additional steam promotes greater hydrogen production. However, when Ca­(OH)_2_ forms (≥40 vol % H_2_ at S/C = 4.5 and for all H_2_ at S/C = 6.5), both CGE and NE decrease sharply, falling below the values at S/C = 2.5. This drop is due to the additional heat required to decompose both CaCO_3_ and Ca­(OH)_2_ during the regeneration stage. Although S/C = 2.5 provides relatively high efficiencies, it also leads to low H_2_ purity (∼95 vol %), highlighting the trade-off between energy efficiency and product quality.

The CO_2_ capture efficiency (Figure 6f) decreases with increasing H_2_ content. This trend is driven by the reduction in CH_4_ conversion with increasing H_2_ concentration, which lowers the amount of CO_2_ generated and, thus, the amount available for capture. Consequently, capture efficiency follows a similar trend to CH_4_ conversion (Figure 6c).

These results highlight the importance of operating conditions that suppress the Ca­(OH)_2_ formation. Since hydration and carbonation compete for CaO and depend strongly on CO_2_ and H_2_O partial pressures, maintaining conditions that favor carbonation is essential to achieving high H_2_ production, effective CO_2_ capture, and robust SESR performance, consistent with the observations of Romano et al.^59^

### 3.3 Maximizing H_2_ Production, CO_2_ Capture, and Energy Efficiency of the SESR Process

This section integrates the preceding results to define the operating window that maximizes the SESR performance. Carbonation, hydration, reforming, and WGS equilibria respond differently to changes in CO_2_ and H_2_O partial pressures. An optimal strategy must simultaneously enhance hydrogen production and CO_2_ capture. It must also avoid the formation of Ca­(OH)_2_, which reduces both the conversion and efficiency.

In the absence of hydration, increasing S/C consistently improves all key performance indicators but at the cost of significant trade-offs. For example, at 10 vol % CO, Ca­(OH)_2_ does not form at any S/C ratio (Figure 4a). At S/C = 2.5, steam is insufficient to drive reforming and WGS, resulting in limited CH_4_ conversion, reduced H_2_ production, and low energy efficiency. Raising the S/C ratio to 4.5 strongly promotes reforming equilibria, achieving near-complete CH_4_ conversion and almost full CO_2_ capture. However, increasing S/C to 6.5 offers only marginal gains despite a much higher steam demand, that is, energy efficiency rises slightly, but the added steam introduces a clear operational penalty. Thus, higher S/C ratios must be weighed against diminishing returns and increased operational costs.

At intermediate compositions, such as 20–30 vol % CO, 10–30 vol % CH_4_, and 0–30 vol % H_2_, Ca­(OH)_2_ remains absent at S/C = 4.5 but becomes significant at S/C = 6.5. In these conditions, S/C = 4.5 offers the best compromise, maintaining high H_2_ production and CO_2_ capture while preserving efficiency by avoiding hydration. For example, at 30 vol % CH_4_, increasing S/C from 4.5 to 6.5 produces only a slight increase in CO_2_ capture, whereas CH_4_ conversion and H_2_ production remain essentially unchanged (Figure 4). Yet, the higher steam demand and additional regeneration energy decrease the net efficiency by 8–10 percentage points. Therefore, the minor capture improvement does not justify the energy penalty, confirming S/C = 4.5 as optimal in this composition range.

For feeds with high CO or H_2_ contents (e.g., 40 vol % CO or 40–50 vol % H_2_), Ca­(OH)_2_ becomes thermodynamically favorable even at moderate S/C ratios. Hydration occurs at both S/C = 4.5 and 6.5. In these cases, performance is better at S/C = 6.5. Despite hydration, the additional steam supply effectively drives reforming. For example, at 40 vol % CO, increasing S/C from 4.5 to 6.5 raises H_2_ purity by ∼2.5 vol % and H_2_ yield by over 9%. Although the efficiency gain is moderate, since the steam requirement counterbalances part of the benefit, the improvement in hydrogen production and CO_2_ capture can justify operation at S/C = 6.5 for these mixtures.

Across all cases, CO_2_ capture efficiency follows a trend similar to that of hydrogen production because both depend strongly on CH_4_ conversion. Higher conversion generates more CO_2_ through reforming and WGS, enabling greater capture. Consequently, avoiding Ca­(OH)_2_ formation is essential not only for sustaining reforming activity but also for enhancing SESR CO_2_ capture.

The interaction between feed composition and process performance is summarized in Figure 7 for S/C = 4.5 and CaO/C = 2.5. In the absence of Ca­(OH)_2_, higher CO contents improve both H_2_ purity and CGE, as CO promotes WGS and lowers the feed CO_2_ partial pressure, enhancing CH_4_ conversion and overall reforming. In contrast, increasing CH_4_ or H_2_ reduces the H_2_ purity because both species suppress the thermodynamic driving force for reforming. Specifically, higher CH_4_ lowers the effective steam-to-CH_4_ ratio at a fixed S/C, reducing its conversion, and H_2_ shifts the reforming and WGS equilibria toward the reactants. However, CH_4_- and H_2_-rich feeds increase CGE because of their higher intrinsic chemical energy, thereby reducing the external fuel required for sorbent regeneration.

**Figure 7:**
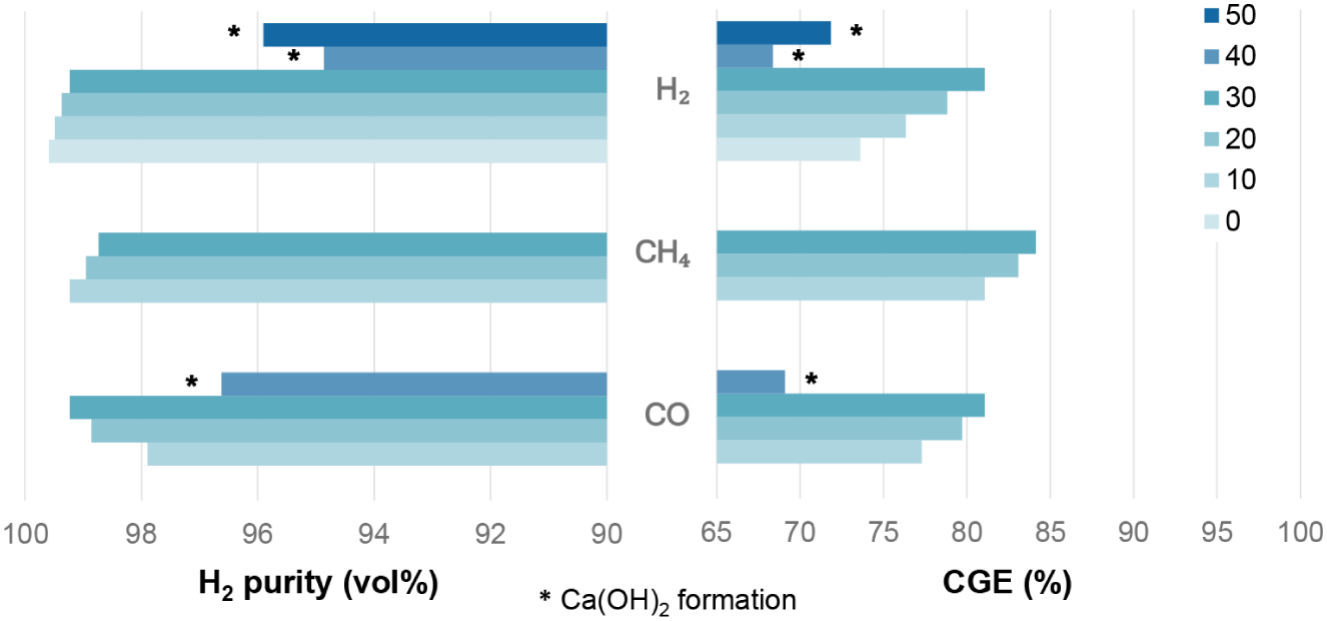
Effect of syngas composition on H_2_ purity and cold gas efficiency (CGE). Simulation conditions: 600 °C, 10 bar, S/C = 4.5, and CaO/C = 2.5. Syngas = 30 vol % H_2_ and 10 vol % CH_4_ for the CO study; 30 vol % H_2_ and 30 vol % CO for the CH_4_ study; and 30 vol % CO and 10 vol % CH_4_ for the H_2_ study (CO_2_ as the balance).

Thus, Figure 7 highlights the inherent trade-off between hydrogen production and energy efficiency.^41^ Specifically, CO-rich feeds maximize H_2_ purity and CO_2_ capture, while CH_4_-rich feeds maximize efficiency due to the higher chemical energy input from methane, but at the expense of slightly lower H_2_ production. For instance, at S/C = 4.5, CGE values above 80% are achieved for feeds containing 30 vol % H_2_ and 30 vol % CO. With 10 vol % CH_4_, H_2_ purity reaches 99.23 vol %, and CGE is 81.06%. Increasing CH_4_ to 30 vol % reduces H_2_ purity slightly (to 98.73 vol %) but increases CGE to 84.13%. Likewise, the highest H_2_ purity (99.59%) is obtained when no H_2_ is present in the feed, but the CGE drops below 75%, demonstrating a significant energy penalty. This comparison clarifies that increasing process efficiency with H_2_-rich or CH_4_-rich feeds results in some reduction in hydrogen purity, while maximizing the purity may incur an energy efficiency penalty. These results show that the presence of H_2_ substantially improves the process efficiency, compensating for its negative effect on purity, which highlights the practical importance of balancing these trade-offs in SESR.

Overall, these results show that the highest SESR performance is achieved at intermediate compositions and moderate S/C ratios, in agreement with previous studies on the SESR of biomass-derived gases, which report maximum hydrogen production when reforming and carbonation are balanced and Ca­(OH)_2_ formation is minimized.^23,24,26,60^ This emphasizes the importance of adjusting operating conditions to expected syngas variability rather than relying on a single fixed set of parameters, enabling robust hydrogen production and high-efficiency CO_2_ capture in practical biomass-derived gas applications.

## 4 Conclusions

This work provides a comprehensive thermodynamic assessment of the SESR of biomass-derived syngas under pressurized conditions, investigating how feed composition (CO, CH_4_, and H_2_) influences hydrogen production, CO_2_ capture, and energy efficiency. By systematically analyzing realistic syngas compositions, the study identifies the key relationships among composition, performance, and energy that govern SESR operation.

The syngas composition strongly influences SESR behavior. CO-rich feeds enhance H_2_ purity, CH_4_ conversion, and CO_2_ capture efficiency by promoting the WGS reaction and favoring methane reforming through reduced CO_2_ partial pressure. In contrast, higher CH_4_- or H_2_-rich feeds limit H_2_ purity and CH_4_ conversion due to reduced effective steam availability and equilibrium inhibition but improve energy efficiency by lowering the external fuel demand required for sorbent regeneration.

Steam availability is a critical operating parameter. Increasing the S/C ratio improves reforming performance up to an optimum, beyond which Ca­(OH)_2_ formation becomes significant, reducing CH_4_ conversion and significantly penalizing energy efficiency due to the additional regeneration heat required. Under optimal conditions (S/C = 4.5, 600 °C, 10 bar), hydrogen purity of 99.2 vol %, CO_2_ capture of 98.8%, and cold gas efficiency of 81.06% are achieved for a representative syngas composition.

Overall, the results define composition-dependent operating windows for pressurized SESR, identifying intermediate syngas compositions (represented by 30 vol % CO, 30 vol % H_2_, and 10 vol % CH_4_) and moderate steam ratios (S/C = 4.5) as optimal for balancing hydrogen production, CO_2_ capture, and energy efficiency. These findings provide practical thermodynamic guidelines and system-level insights for the development of flexible, energy-efficient SESR systems capable of processing variable biomass-derived syngas, thereby supporting their integration into low-carbon biorefineries.

## Supplementary Material



## Notes

Aspen Plus flowsheet for sorption-enhanced steam reforming of syngas and results on the effect of feed composition on H_2_ yield, CO conversion, and outlet CH_4_ concentration (PDF)
