# Supplementary material for: Autothermal Sorption-Enhanced Steam Reforming of Renewable Syngas: Composition-Dependent Hydrogen Yield and Energy Efficiency
Source: Energy Fuels. 2026 Jul 17;40(30):16757–69. doi: 10.1021/acs.energyfuels.6c01902 (PMC13435604; doi:10.1021/acs.energyfuels.6c01902)
Supplement: Supplementary file 1 [file ef6c01902_si_001.pdf]

# *Supporting Information*

# Autothermal Sorption-Enhanced Steam Reforming of Renewable Syngas: Composition-Dependent Hydrogen Yield and Energy Efficiency

Alejandra Vega, Fernando Rubiera, Covadonga Pevida, María Victoria Gil

*Institute of Carbon Science and Technology (INCAR), CSIC, Oviedo 33011, Spain*

# 1. Effect of CO content in syngas

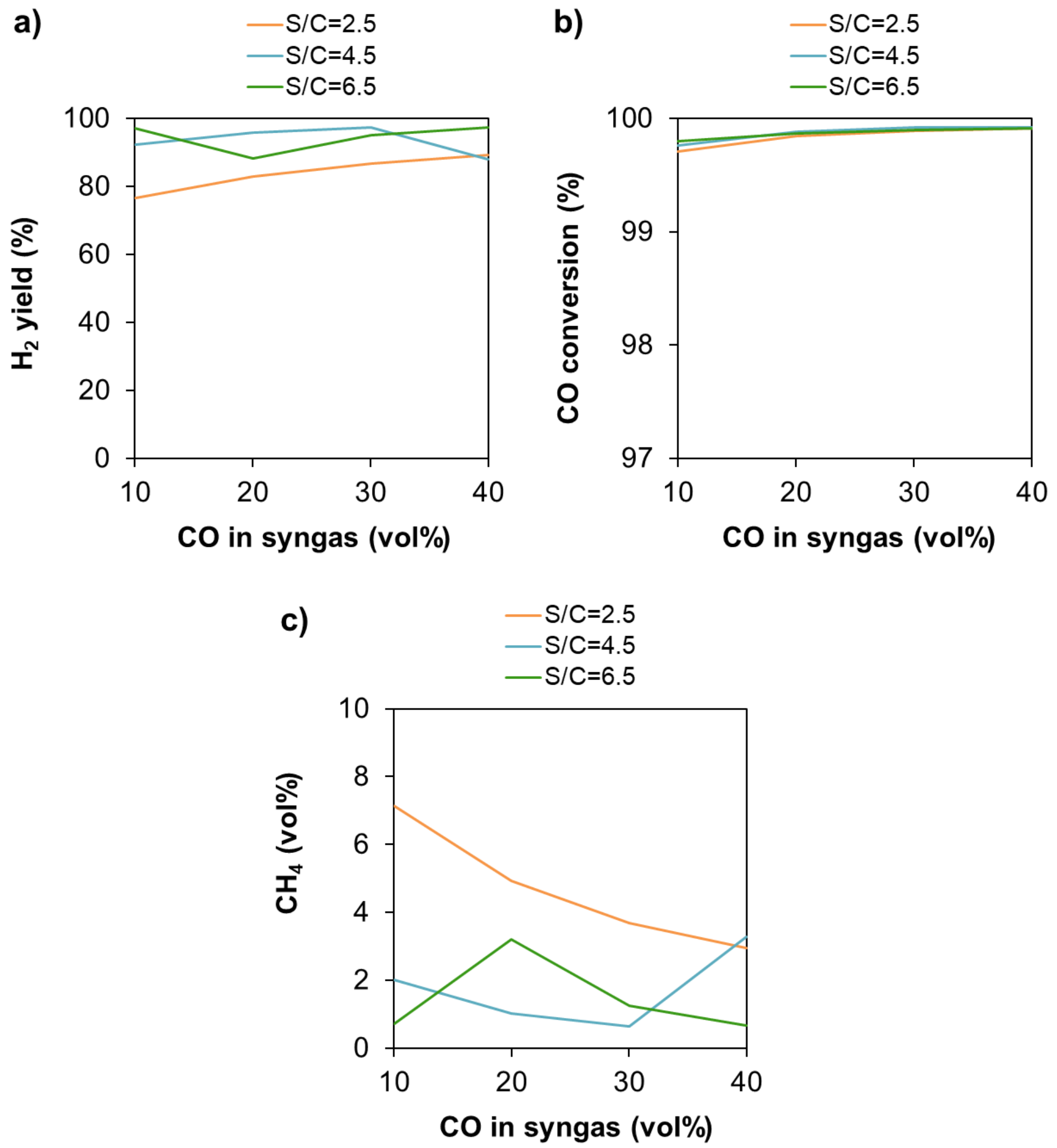


**Figure S1.** Effect of CO content in the syngas on SESR performance: (a) $H_2$ yield, (b) CO conversion, and (c) $CH_4$ concentration in the outlet gas from the SESR reactor. Three steam-to-carbon ratios (S/C = 2.5, 4.5, 6.5) are compared. Conditions: 600 ºC, 10 bar, CaO/C = 2.5, syngas = 30 vol% $H_2$, 10 vol% $CH_4$, 10–40 vol% CO, and $CO_2$ as the balance.

## 2. Effect of $CH_4$ content in syngas

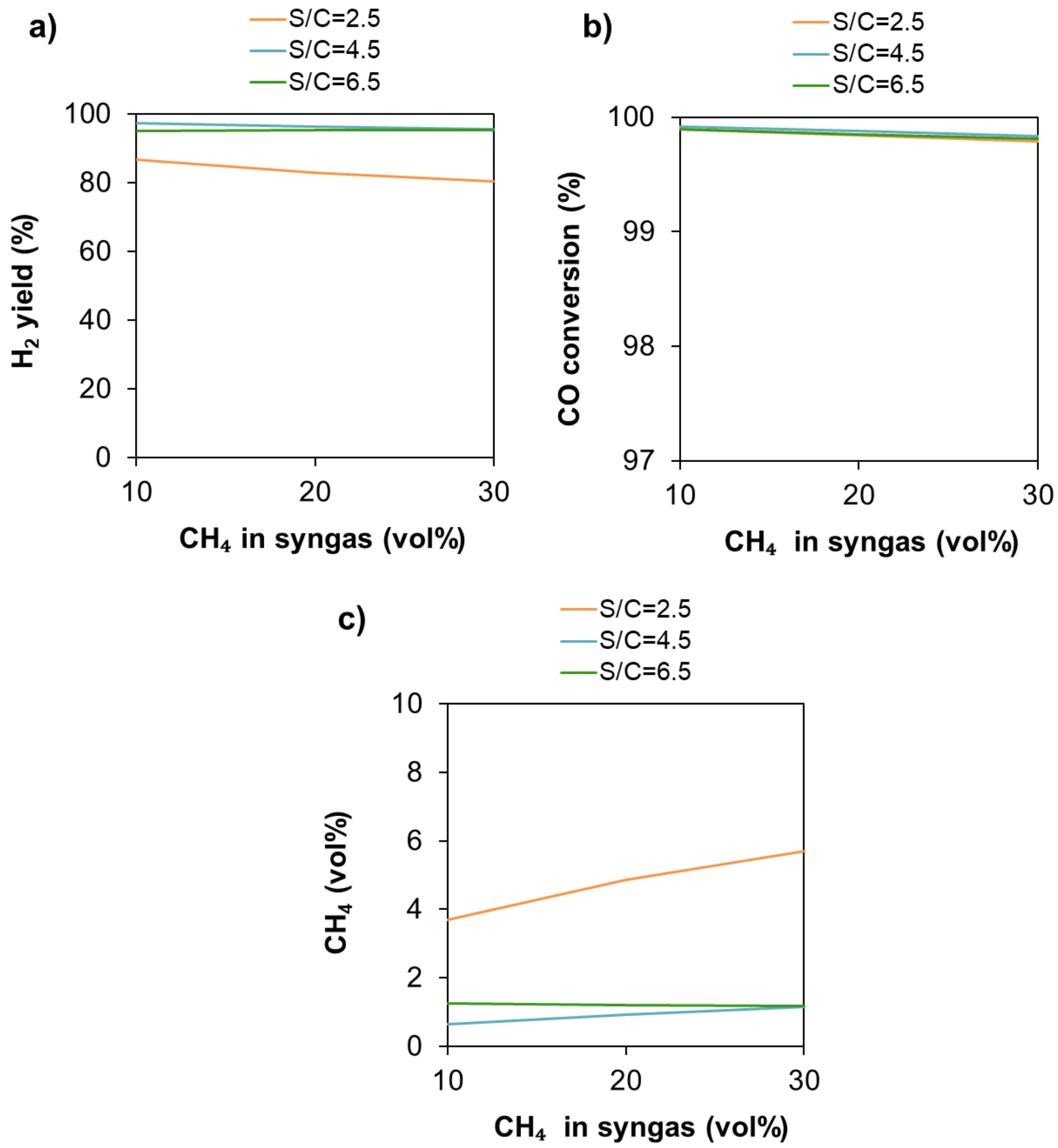


**Figure S2.** Effect of $CH_4$ content in the syngas on SESR performance: (a) $H_2$ yield, (b) CO conversion, and (c) $CH_4$ concentration in the outlet gas from the SESR reactor. Three steam-to-carbon ratios (S/C = 2.5, 4.5, 6.5) are compared. Conditions: 600 ºC, 10 bar, CaO/C = 2.5, syngas = 30 vol% $H_2$, 30 vol% CO, 10–30 vol% $CH_4$, and $CO_2$ as the balance.

## 3. Effect of $H_2$ content in syngas

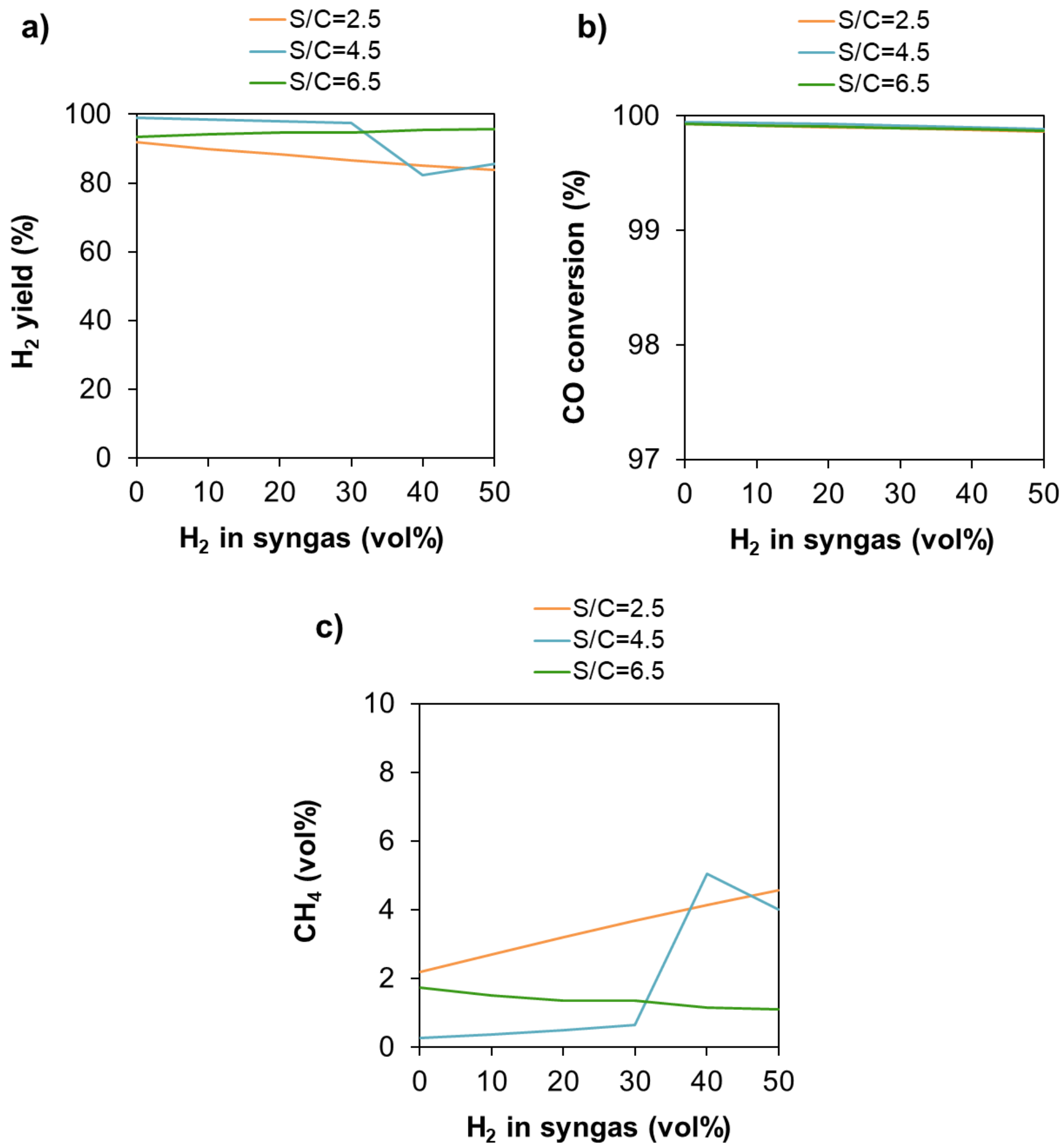


**Figure S3.** Effect of $H_2$ content in the syngas on SESR performance: (a) $H_2$ yield, (b) CO conversion, and (c) $CH_4$ concentration in the outlet gas from the SESR reactor. Three steam-to-carbon ratios (S/C = 2.5, 4.5, 6.5) are compared. Conditions: 600 ºC, 10 bar, CaO/C = 2.5, syngas = 30 vol% CO, 10 vol% $CH_4$, 0–50 vol% $H_2$, and $CO_2$ as the balance.
